# Uvula Manipulation and Resonance (UMAR) Treatment for Puberphonia

**DOI:** 10.1007/s12070-021-02412-3

**Published:** 2021-03-09

**Authors:** Muthiah Kumaresan, Navin Bharath Kumaresan, Parameswaren Darling Elangovan

**Affiliations:** 1 Department of Otorhinolaryngology, Siva ENT Hospital, 295, Triplicane High Road, Triplicane, Chennai, India; 2 Department of Otorhinolaryngology, Saveetha Medical College, Chennai, India; 3 Siva ENT Hospital, Chennai, India

**Keywords:** UMAR, Uvula manipulation, Voice resonance, Puberphonia, Speech problem, Ancestral voice

## Abstract

Otolaryngologists and speech therapist can be experts in managing specific puberphonia lesions to improve voice. However, not all voice problems have lesions amenable to surgical or medical therapies. Many are associated with maladaptive speech behaviors. We may employ a variety of techniques to improve vocal quality and function in patients with and without structural or neurologic laryngeal pathology. There is an alarming increase in cases of puberphonia and it’s after effects. Otolaryngologists can partner to manage a constellation of puberphonia voice problems with directed voice evaluation and therapy. We, in a small Otorhinolarygolist center in a small city, Chennai has registered and treated 600 cases of puberphonia. We are able to register the devastating problems of puberphonia. Regarding the treatment of puberphonia, in which laryngeal massage, outside the neck, is consistently highlighted as being an effective therapy technique for treating it. In addition, there have been several individual studies conducted to examine the effectiveness of laryngo pharyngeal manipulation inside the vocal tract, which also yielded positive results. A basic review of UMAR techniques and this partnership with yoga breathing trainers is presented which give almost immediate low pitch speech and give a good follow up.

## Objectives

We aim to study the efficacy of voice changing from high pitch to low pitch and maintain the voice at low pitch for ever in puberphonia patients. The objective of the study was to validate the use of a customized voice therapy program for puberphonia patients based on comprehensive voice assessment and behavioral therapy techniques. Voice therapy for puberphonia is a promising modality of treatment but take long time for 78.9% success in 3 to 6 month time therapy. Vocal cords manipulation/vocal cord crushed with the help of laryngeal forceps/under GA with 83.33% success. Types 3 thyroplasty surgery tried in failed voice therapy [8, 9]. Our new UMAR training in the treatment of puberphonia is similar to voice therapy. Instead of external voice therapy or external conventional laryngeal manipulation, we do internal pharyngeal manipulation and resonance at uvula level and get the desired permanent result early [11].

## Introduction

Be prepared for immediate results in puberphonia treatment by uvula manipulation and habituate it by voice resonance. One of the treatments for puberphonia is direct voice therapy. Techniques used include: (1) Cough: The patient is asked to apply pressure on the Adam's apple and cough. (2) Put a tongue depressor and examine the throat and patient is asked to say few words [8, 9]. (3) While doing endoscope patient is asked to say few words. (4) Patient is asked to snore, much parent report that their children’s are snoring like an adult male. This results in the change in the air flow and resonance, which is the physiological mechanism that reduces pitch. The patient can practice continuously by uvula resonance with forcible air flow from stomach/diaphragm. The voicing can be practiced at the level of uvula by a lower pitch continuously. Yes, we can create and give immediate result in puberphonia voice change, but we have to maintain it and make it as a habit [2].

## Case Study

Why more male not taking treatment for puberphonia.

Recently, I saw a young man, 25 years old, who presented with a very high pitched voice. Unlike most puberphonics, he was not particularly embarrassed about his voice but he was aware that the pitch was high, that his voice fatigued easily and that he could not project his voice. As with many handicaps, there are some misconceptions about puberphonia [1]. A common myth is that it’s simply a result of somebody being overly nervous and shy. While having a puberphonia may cause someone considerable anxiety in social situations, such anxiety itself does not cause puberphonia but puberphonia result in anxiety. Much particularly reserved type of people may be puberphonic, because they never want to exhibit theirvoice [3]. Additionally, it is usually not the result of childhood trauma as some believe like bad parenting. Unusually, he could produce a lower pitched voice with difficulty but he felt both physically and psychologically uncomfortable in the lower voice [6]. I asked what his friends thought about his higher voice and he said that they were used to it. The real issue was that he was used to his higher voice. He considers this voice tone is a symbol for him to be recognized by others.

We humans tend to be most comfortable with what we are used to even if what we are used to is inappropriate or limiting for us. Have you ever come across a person, especially a male person having a female-like voice or childish voice, and worse still, have you ever bullied or made a bad joke for his having such a mismatched adolescent voice? [7] This is the peer influence. The person might be suffering from a more serious medical mental inferiority complexity. Don’t make it so difficult for the person; he is already having a hard life with depression! And if you are seeing and hearing, the one suffering from such a voice disorder, and is facing such awkward situations in your day-to-day life, then you are probably suffering from Puberphonia. Do not panic, as it can be cured completely [10]. Give a confidence and treat it immediately. With an attractive voice, which everyone would love to hear? No problem with sexual activity, family life and can get married. Confidently conform that they will have children.

## Method

### Material and Description of Tool

The study was carried out as a descriptive simple purposing sampling technique. A self administered questionnaire on impact of puberphonia in the society was used. Variables such as age, gender, religion, sex, birth order, educational qualification, occupation, income marital status and personal address proof such as phone number, e-mail id, and questions rose in order to assess the impact of puberphonia difficulty in talking to girls, difficulty in way of communication, psychological distress, different copying, family history, emotional status, etc.

### Selection and Description of Participants

**Fig. 1:**
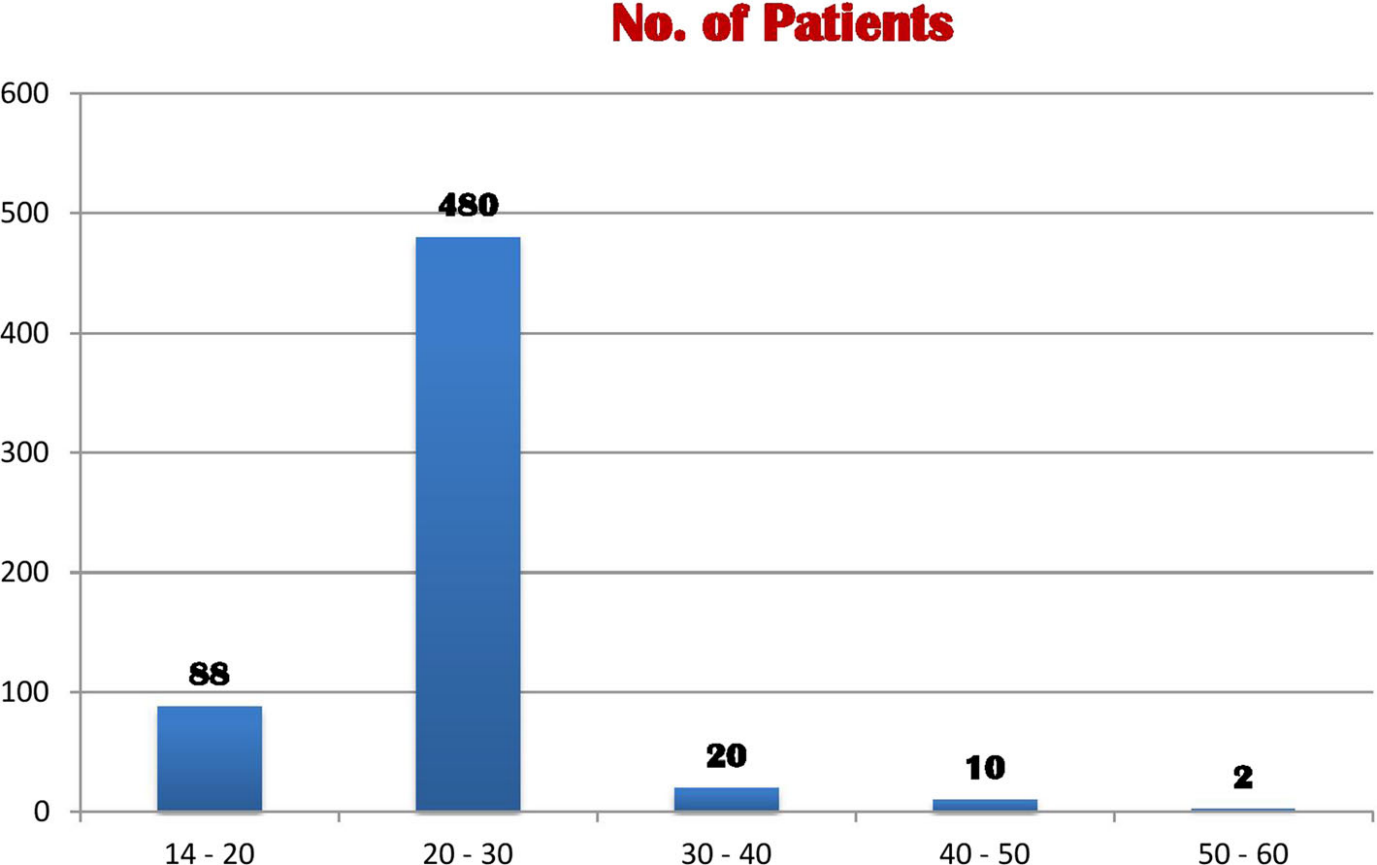
Age-wise number of Patients

The target population of study comprised of age groups 15 years to 70 years (Fig. 1). Sample selection criteria were based on who ever willing to participate in the study. Study sample were selected by using convention sampling technique based on sample selection criteria.

### Data Collection

**Fig. 2:**
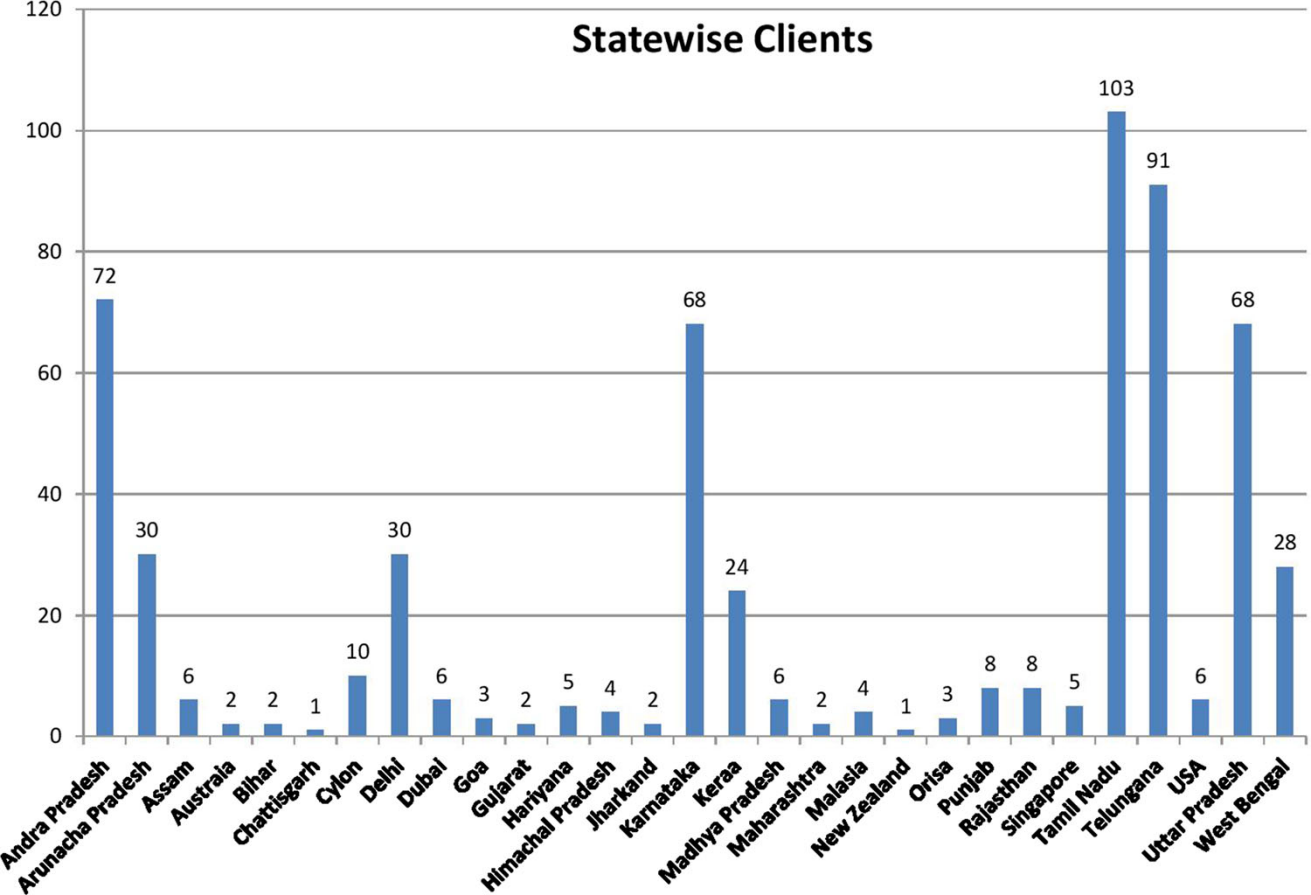
Statewise number of Patients

After getting the written permission from each sample, the structured questionnaire was used to assess the impact of puberphonia on society by interview method (Fig. 2). During the time of initial period some boys have shared their feelings like drop-out of their studies, not getting good job, not interested in marriage, (although they are willing for marry and their testosterone level is in normal male range, they reluctant to get marry due to their female phonation). They feel shame and guilt in all walks of their life and they looked in a much despaired state, some boys have gone to the extent that, they would have commit suicide.

### Treatment Method

It is divided in to 5 parts.

**Fig. 3:**
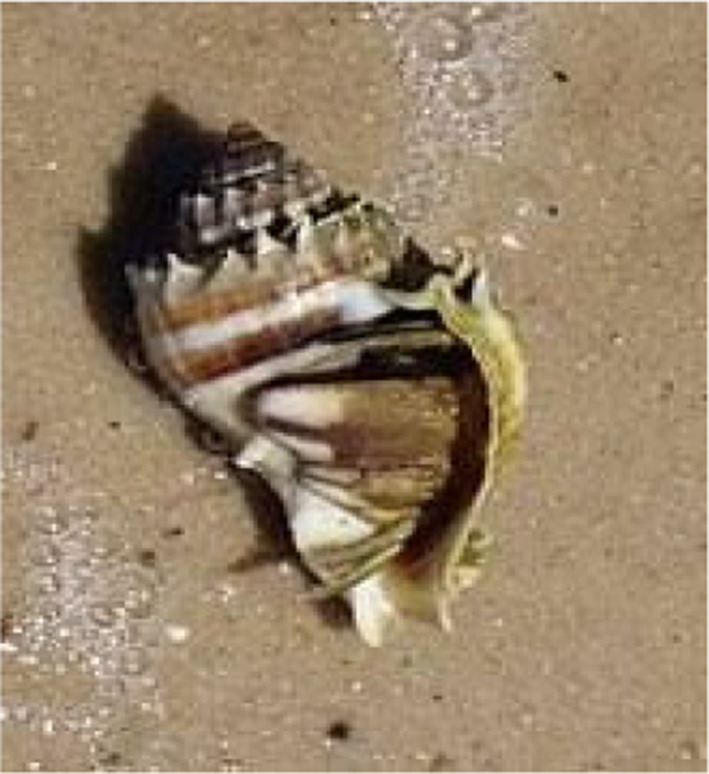
Sanku

**Fig. 4:**
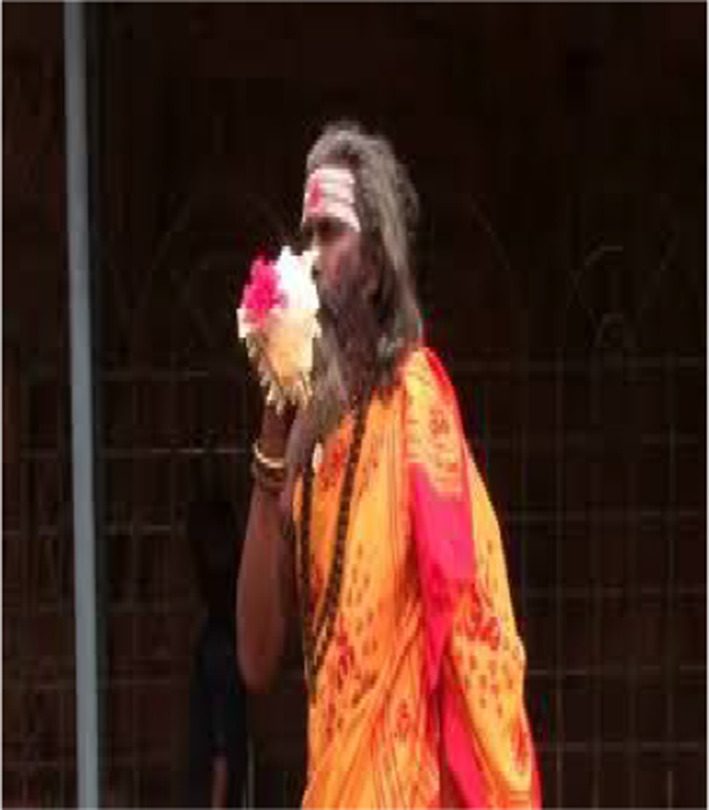
Sakku blowing/“Breath of fire”

**Fig. 5:**
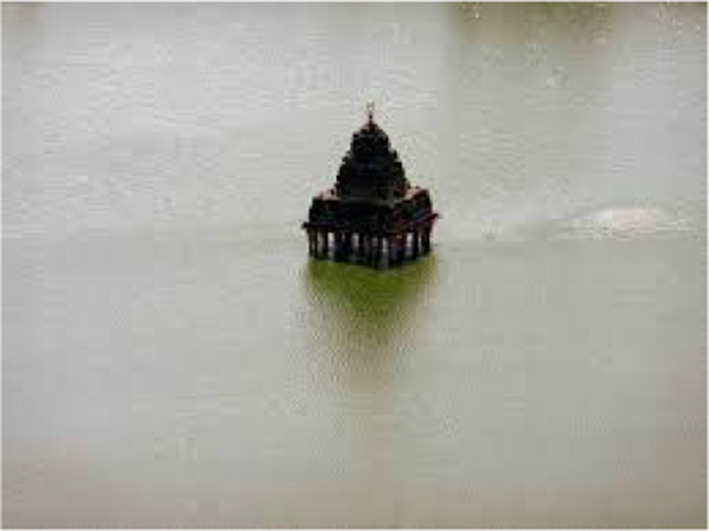
Sanku mandabam in river

1. Treatment n the minor operation theater itself.
2. Three days continuous breath of fire training in a group therapy (Figs. 3, 4 and 5).
3. Home training with regular contact with senior treated puberphonia boys at SIVA ENT hospital for 21 days.
4. Every Saturday 3 pm virtual online meeting and online updating about puberphonia.
5. Every year 4 times we conduct one full day work shop on puberphonia.

The method reported by the author; while doing examination of the pharynx by tongue depressor or direct laryngoscope examination of a patient of puberphonia few are able to bring out normal male voice, while they cough, yarn, snore or talk. We have used a novel approach by UMAR which gives immediate and permanent relief. Puberphonia patients underwent detailed ENT evaluation by an ENT surgeon and a stroboscopic evaluation was done using Kay Pentax 9105 System. Stroboscope was used to obtain a visual assessment of the vocal cords. A perceptual assessment of patient’s voice was done using the GRBAS scale. This scale consists of judgment of voice quality on the basis of Grade (G), Roughness (R), Breathiness (B), Asthenia (A), and Strain (S) in voice production. Patient was called nil by mouth six hours before the procedure. The selected case is taken to the minor operation theatre. Procedure was done in the supine position. Under xylocaine (10% w/v) spray surface anesthesia a silk thread is placed in the uvula by suturing or knots. The ENT Surgeon himself performs and demonstrates the fire of breath breathing technique in the theatre itself. The main role of the respiratory component in sound production is to produce an outward flow of air under pressure. The /k/sound is made through the mouth and it is unvoiced which means that you don’t use your vocal cords to make the sound.

Training to habituate the usage of low pitch voice; It is defined by position of your tongue and it is a stop sound, which is a sound made by building up air pressure by stopping air flow and then releasing it. We give group voice breathing training as it is a stimulating factor with their puberphonia treated colleagues. It will give a fun with fire of breathing. They may express their story telling in the event of group training practice. Group therapy encourages, I-relaxing and focusing on lessons taught, accidental, enjoyable on posture and breathing, II-preparing to do with phonation by uvula manipulation, III-finding the target voice by them self, IV-advance using the new phonation style voicing-adopting the new muscle technique to speak with good resonance. It results in lengthening the phonatory apparatus and widens the vocal tract and training abdomino diaphragmatic respiration.

### Recurrent Rate

**Fig. 6:**
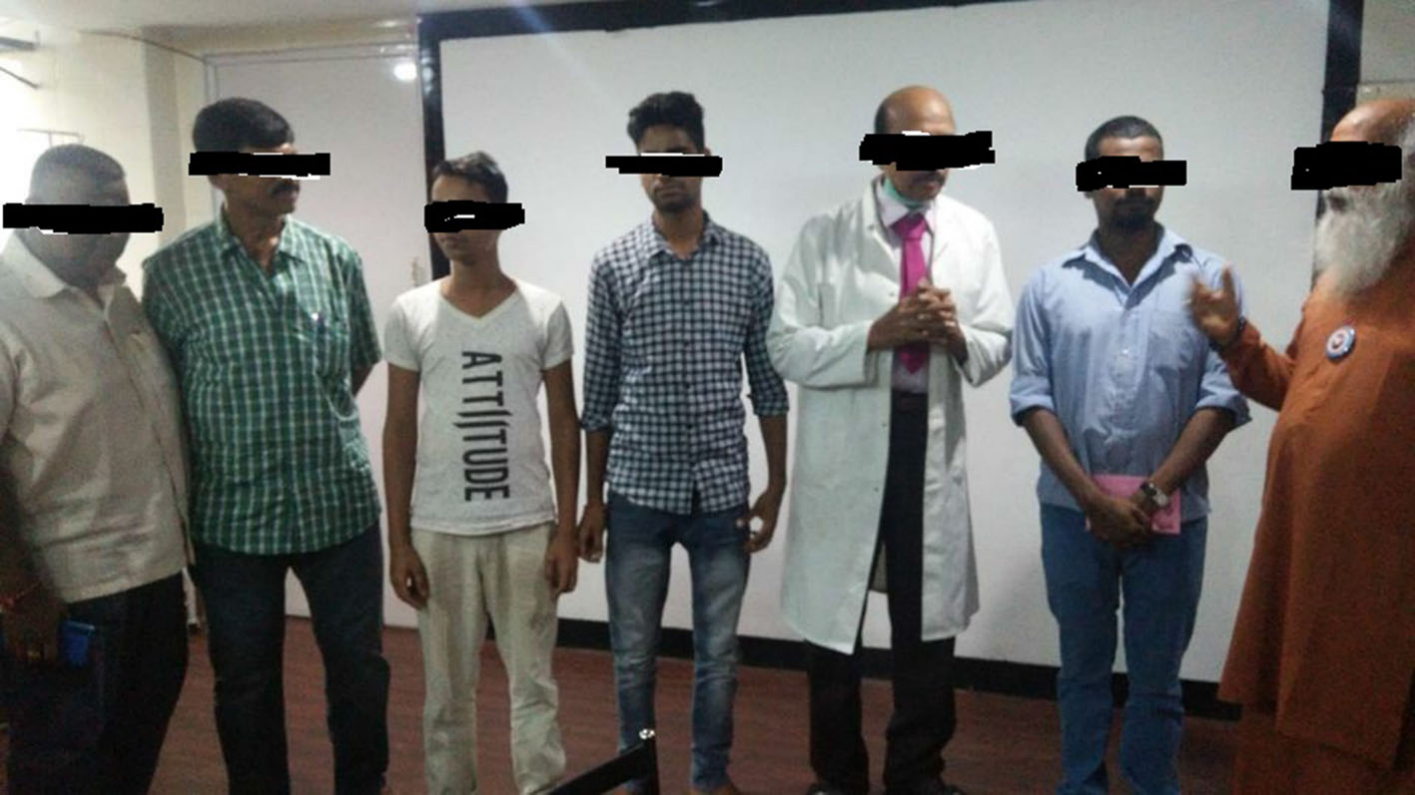
Group therapy

**Fig. 7:**
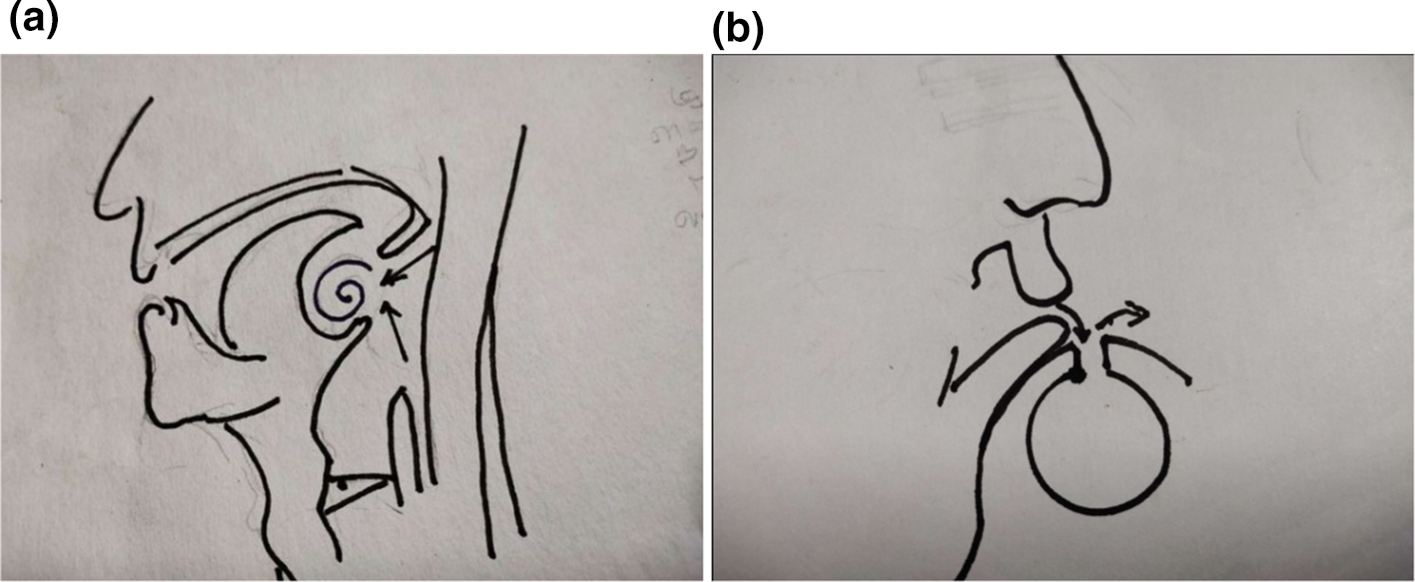
Air forcibly moves at the uvulopharyngeal complex (both Bernoulli’s and Venturi effect works), similar to lip air blowing vocal musical instruments- flute

**Fig. 8:**
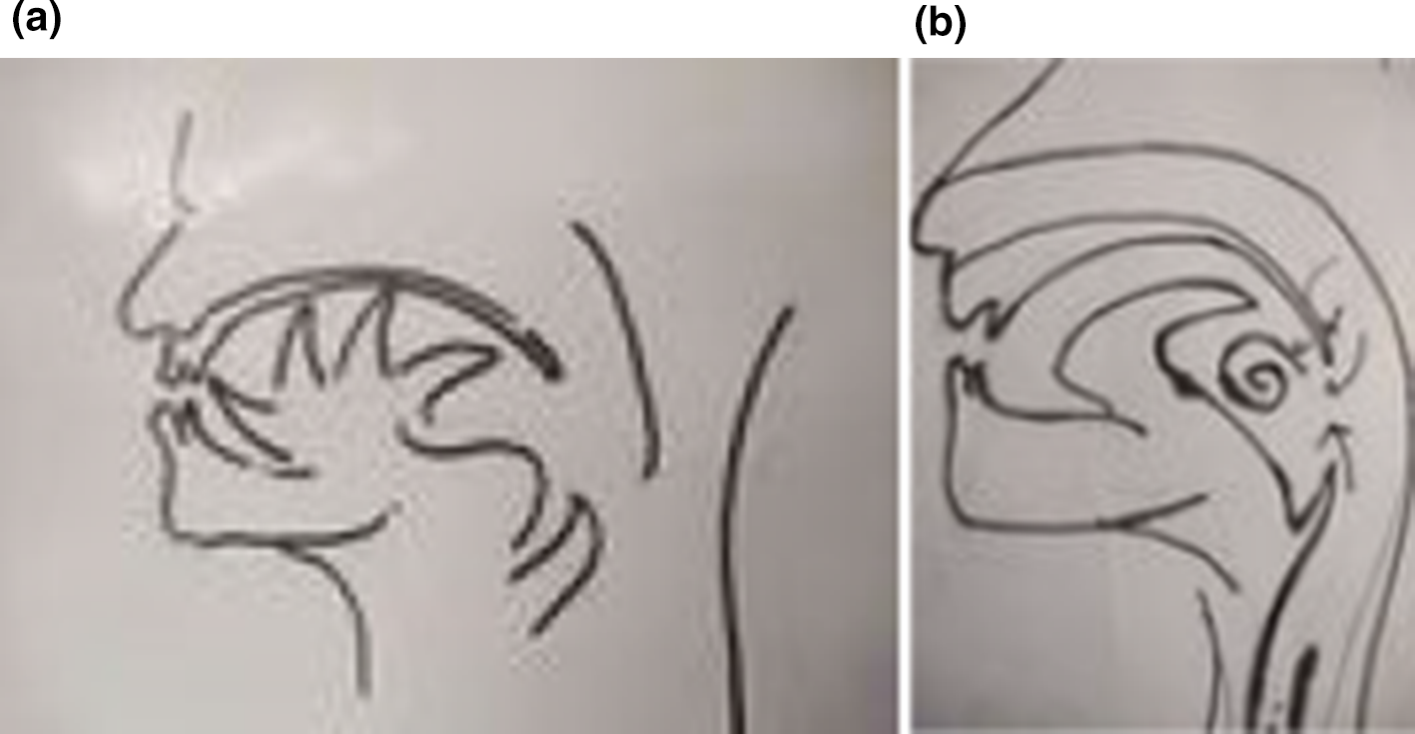
Movement of tongue in palatal and uvular consonants

We periodically give reminder calls as well as do follow-up fifth, 21 days, 3 months, 6 months and one year. We analyze the frequency, and as such we found almost all persisted male voice, but 12.5% cases voice regressed back due to not following the exercise we advised. For them we call to our Puberphonia Institution for further management. In this recurrent program for 3 days most of them resumed male voice by intensive voice therapy. 10% of the cases continue to have high pitch voice then and there, may be due to unknown facts (Figs. 6, 7, 8).

These figures shows number of the clients underwent UMAR training in Siva E N T Hospital at Puberphonia clinic (Figs. 12 and 13).

## History of Puberphonia Treatment in Our Hospital

**Fig. 9:**
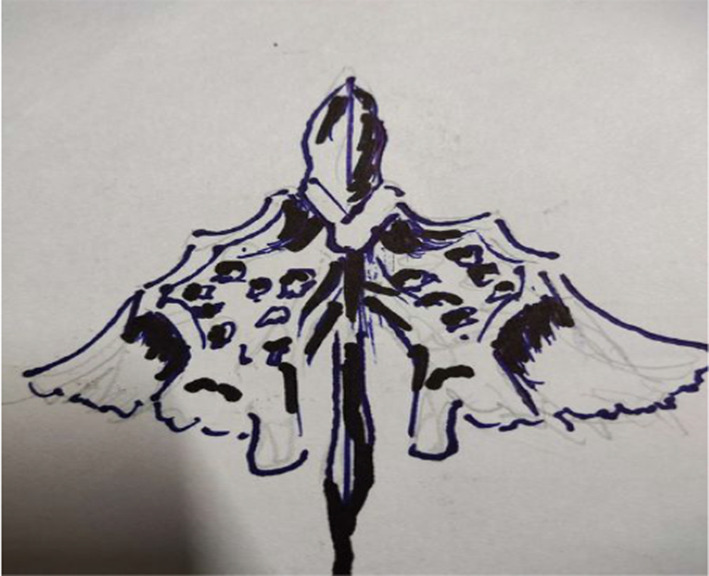
Labyrinthine resonance

Being an E N T Surgeon I am treating many diseases and disorders pertain to my specialty, but I come across puberphonia 15 years back did research and found treatment methods in Tholkappiam (the Epic in Tamil) in that book the treatment Technique is explained. Our therapy for puberphonia is based on Tholkappiyar (The scholar in Tamil language, 1st–fourth century ce; “Ancient Literature”, is a treatise on grammar and poetics) [5]. In his poem on the birth of speech, he had given an inspiration for our work, uvula manipulation and resonance training. This work on the grammar reveals that the speech originate from the stomach, divert and resonate from the level of oropharyngeal junction (Fig. 9). Then onwards we are doing this therapy successfully to all puberphonia clients. This minor manipulation is done at the uvula and followed by breath of fire breathing training. On the first day it-self many resume lower pitch voice and also seen some progress on other variability like splitting of the voice, dysphonia and cutting of the voice and etc. We give three to five days resonance therapy and their frequency and other variability are analyzed daily and compare with previous frequency, cutting of voice, whether breathy voice accomplishing on phonation and presence and absence of dysphonia. Many boys resume successful result on the third day onwards, some gets on 5th day and for very few, it may take week or ten days. On the day of discharge we tell about the follow-up exercise program. We give exercise format, we do follow-up on 21 days, 3 months 6 months and 1 year.

Out of 600 cases, before uvula manipulation and resonance therapy, most of the clients present with following symptoms like the range of frequency would be 200 to 265 Hz, cutting of voice, splitting of the voice, female voice on phonation and dysphonia. Whereas on the first day of the manipulation therapy itself, 350 of them got “the transition on other variable” and on the third day, it progressively regress all symptoms. At the same time, out of remaining 50 cases 30 patients recovered to male frequency on fifth day. Whereas for 20 patients voice frequency remained in the range of 190 to 200 Hz and partially persisted other symptoms. For them also we give intensive speech therapy for 10 to 30 days. Most of them progressed well, but 10 patients resist changing their voice in spite of the effort we put, probably, I think, due to some other unknown pathology.

### Recurrent Rate

We periodically give reminder calls as well as do follow-up fifth, 21 days, 3 months, six months and one year. We analyze the frequency, and as such we found almost all persisted male voice, but 2.5% cases voice regressed back due to not following the exercise we advised. For them we call to our Puberphonia Institution for further management. In this recurrent program is for 3 days. Still 10 patients are not interested in continuing the program and we believe them as dropout.

## Discussion

**Fig. 10:**
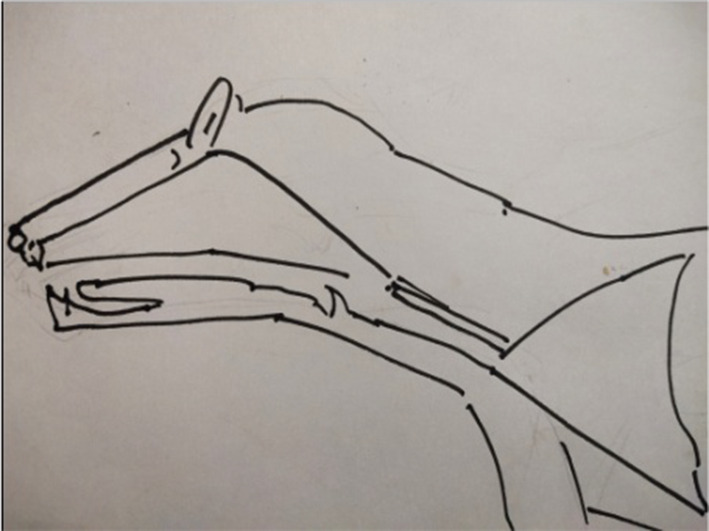
Animal: straight vocal tract and no uvula (Animals: no uvula)

**Fig. 11:**
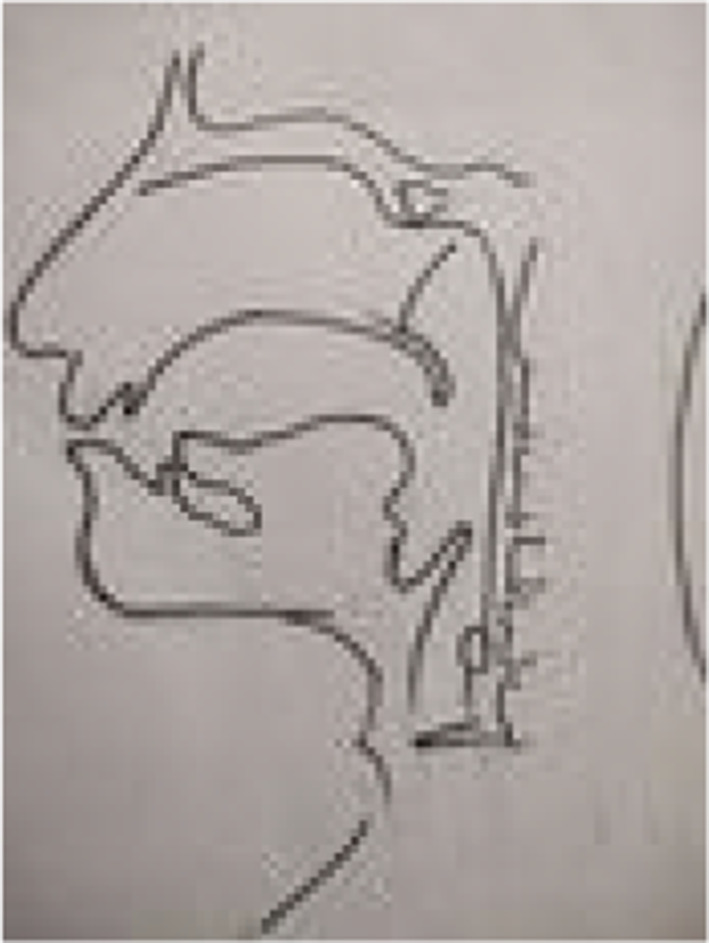
Human: the two halves of tongue meet at an approximate right angle at the back of the throat (Human: possess uvula and two portion in tongue: Horizontally in the oral cavity and vertically in the pharynx)

**Fig. 12:**
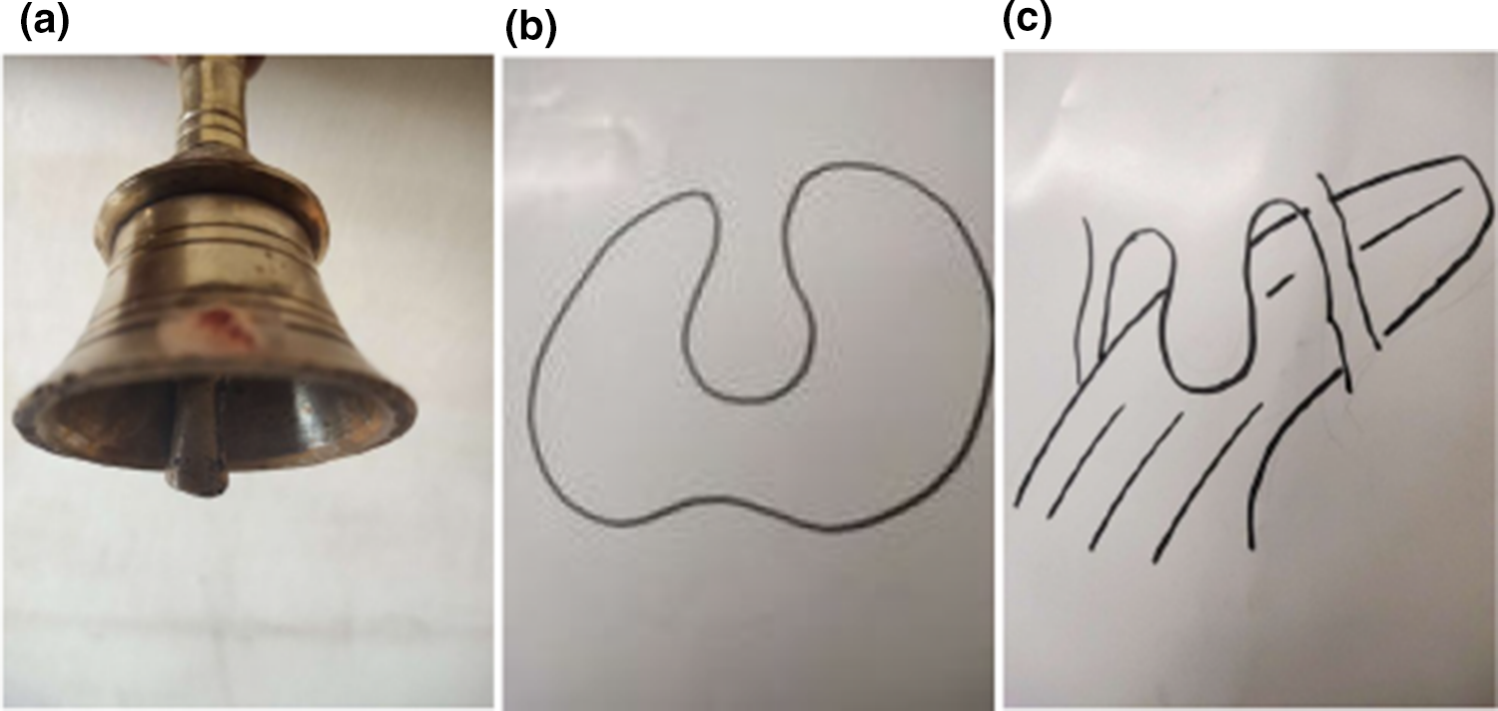
Uvula position/action is comparable to a bell

**Fig. 13:**
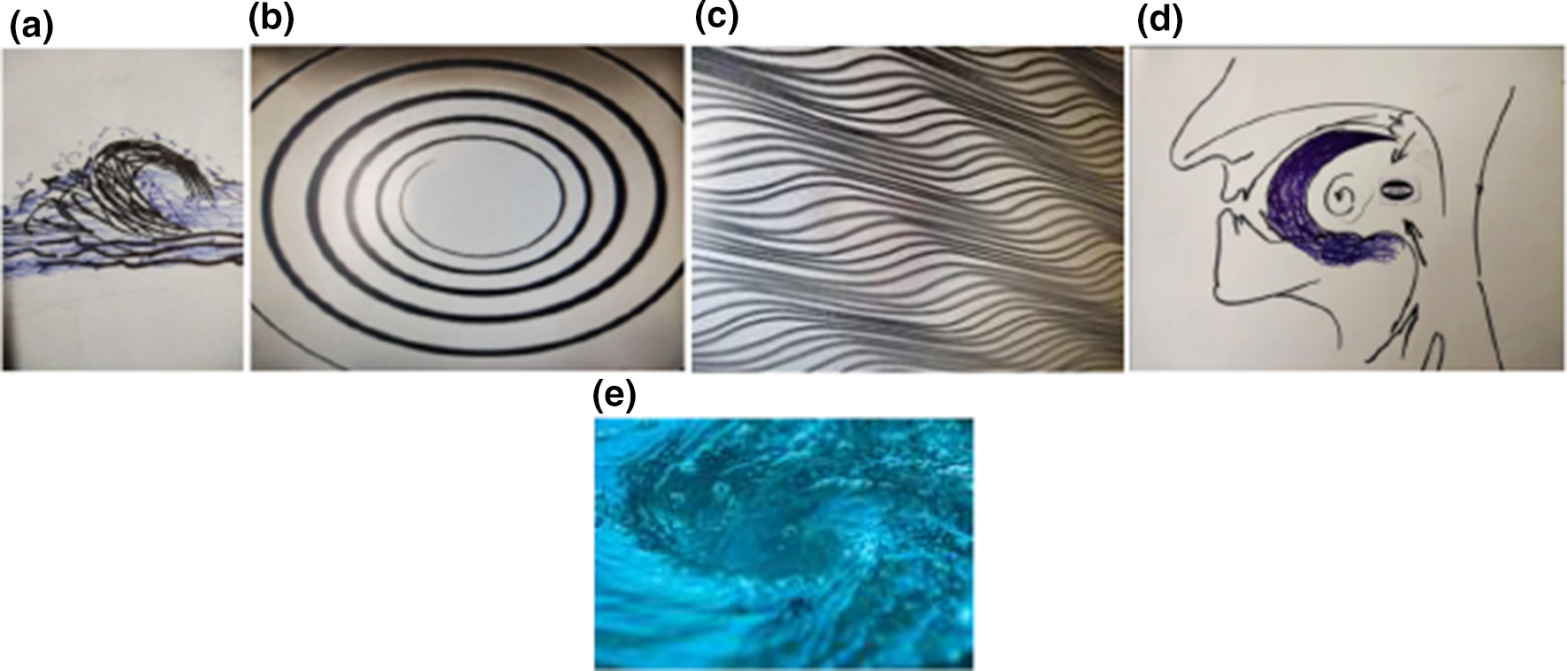
Demonstration of Ripple effect  and vortices

From 1990 onwards we have done trying various methods to treat puberphonia. Earlier days laryngeal stretching was done by us with Rush-Miller laryngoscope and we published it in our book “A research in otolaryngology". There was an immediate improvement in voice quality from child pitch to male pitch. Patients were followed and had been sent to speech therapist. There was no consistent improvement in the voice quality to male pitch. He did it repeatedly. In this article Dr. Sudhakar Vaidya [13] stated "no reference is available except from Dr. M. Kumaresan (Chennai)", who has published his work in book "a research work in Otolaryngology" in 1992 [12]. Both uvula and speech serves to differentiate human beings from animals (Figs. 10 and 11). Uvula position, movement, secretion and action (Fig. 12a, b and c)  play a major role in augmenting the force of the air filled with sound, which is capable of producing more energy in voice production. Uvula moves vibrate increasing size and by its abundance salivary secretion keep the pharynx moist and well lubricated. Contraction of the musculature uvula adds the bulk is reported (Fig. 13a, b, c, d and e). Previous researches indicated the palate/uvula does only on–off function in speech production. It is suggested that palatal /uvula musculature would be predicted to contract more forcefully when necessary/to produce greater intra oral pressure and require for greater degree of oral tract construction required for voice production [4]. In this paper we present a description of uvula function during pharyngeal/uvula resonance manipulation for treating puberphonia. These observations are considered preliminary and are subject to revision as the data from all subjects are analyzed in great details with more refined methods.

## Conclusions

As this uvula manipulation and resonance procedure needs no surgery it is cost effective and no post treatment complication. The surgeon who performs the procedure and the patient recognize the desired voice immediately after this simple manipulation procedure in some puberphonia treated clients. Added to that, few boys immediately recognize the new voice which is similar to their ancestral voice. In such cases immediate phone by the puberphonia treated boys to their family members create a climax in our procedure. Relatives and friends also recognize and appreciate them. This study was carried out with the goal of documenting the efficacy of voice therapy by UMAR in terms of lowering pitch as well as documenting the effect of voice therapy on quality of life in persons with puberphonia. This study is conducted and review systematically the literature and to analyze the effectiveness of UMAR therapy in addressing the overall severity of puberphonia disorder. The study suggests that the UMAR therapy may be considered as an essential mode of treatment for puberphonia.

When we combine that knowledge with what I hear in the speech tone and with the behavior of the puberphonia, my ability to figure out the true cause of the problem grows massively, is that puberphonia is not a disease, but it is a life style. That can only mean that my clients are able to overcome blocks and develop even quicker, which ultimately benefits their careers and well-being of the society.

## Recommendations

1. The study can be done with rural and urban puberphonia males.
2. The comparative study of impact of puberphonia among adolescent and adults.
3. The study can be done to assess the other problem of puberphonia.

## Appendix

Puberphonia/health questionnaire-too used.
